# iPhone Sensors in Tracking Outcome Variables of the 30-Second Chair Stand Test and Stair Climb Test to Evaluate Disability: Cross-Sectional Pilot Study

**DOI:** 10.2196/mhealth.8656

**Published:** 2017-10-27

**Authors:** Gautam Adusumilli, Solomon Eben Joseph, Michael A Samaan, Brooke Schultz, Tijana Popovic, Richard B Souza, Sharmila Majumdar

**Affiliations:** ^1^ Musculoskeletal Quantitative Imaging Research Group Department of Radiology and Imaging University of California San Francisco San Francisco, CA United States; ^2^ Human Performance Center Department of Orthopaedic Surgery University of California San Francisco San Francisco, CA United States; ^3^ Human Performance Center Department of Physical Therapy and Rehabilitation Science University of California San Francisco San Francisco, CA United States

**Keywords:** osteoarthritis, telemedicine, mobile phone, mobile apps, algorithms, medical informatics

## Abstract

**Background:**

Performance tests are important to characterize patient disabilities and functional changes. The Osteoarthritis Research Society International and others recommend the 30-second Chair Stand Test and Stair Climb Test, among others, as core tests that capture two distinct types of disability during activities of daily living. However, these two tests are limited by current protocols of testing in clinics. There is a need for an alternative that allows remote testing of functional capabilities during these tests in the osteoarthritis patient population.

**Objective:**

Objectives are to (1) develop an app for testing the functionality of an iPhone’s accelerometer and gravity sensor and (2) conduct a pilot study objectively evaluating the criterion validity and test-retest reliability of outcome variables obtained from these sensors during the 30-second Chair Stand Test and Stair Climb Test.

**Methods:**

An iOS app was developed with data collection capabilities from the built-in iPhone accelerometer and gravity sensor tools and linked to Google Firebase. A total of 24 subjects performed the 30-second Chair Stand Test with an iPhone accelerometer collecting data and an external rater manually counting sit-to-stand repetitions. A total of 21 subjects performed the Stair Climb Test with an iPhone gravity sensor turned on and an external rater timing the duration of the test on a stopwatch. App data from Firebase were converted into graphical data and exported into MATLAB for data filtering. Multiple iterations of a data processing algorithm were used to increase robustness and accuracy. MATLAB-generated outcome variables were compared to the manually determined outcome variables of each test. Pearson’s correlation coefficients (PCCs), Bland-Altman plots, intraclass correlation coefficients (ICCs), standard errors of measurement, and repeatability coefficients were generated to evaluate criterion validity, agreement, and test-retest reliability of iPhone sensor data against gold-standard manual measurements.

**Results:**

App accelerometer data during the 30-second Chair Stand Test (PCC=.890) and gravity sensor data during the Stair Climb Test (PCC=.865) were highly correlated to gold-standard manual measurements. Greater than 95% of values on Bland-Altman plots comparing the manual data to the app data fell within the 95% limits of agreement. Strong intraclass correlation was found for trials of the 30-second Chair Stand Test (ICC=.968) and Stair Climb Test (ICC=.902). Standard errors of measurement for both tests were found to be within acceptable thresholds for MATLAB. Repeatability coefficients for the 30-second Chair Stand Test and Stair Climb Test were 0.629 and 1.20, respectively.

**Conclusions:**

App-based performance testing of the 30-second Chair Stand Test and Stair Climb Test is valid and reliable, suggesting its applicability to future, larger-scale studies in the osteoarthritis patient population.

## Introduction

Osteoarthritis is the most prevalent chronic condition of the joints, affecting approximately 30 million Americans and one in every two adults during their lifetimes [1]. Osteoarthritis most commonly occurs in the weight-bearing hip and knee joints and is the consequence of a progressive breakdown process of articular cartilage, joint capsule, and ligaments; synovial tissue inflammation; and subchondral bone sclerosis [2-5]. Patients diagnosed with osteoarthritis experience a decline in physical function and often report difficulty performing activities of daily living (ADL) [6]. Recent work has shown that osteoarthritis patients place increased compensatory stress on their unaffected weight-bearing joints during ADL, a vicious cycle that makes them susceptible to further damage and worsened severity of disease [7-9].

It is imperative for the development of a treatment plan to identify osteoarthritis-induced deteriorations in physical function and each patient’s respective compensatory mechanisms. In 2013, a battery of five tests simulating ADL was recommended by the Osteoarthritis Research Society International (OARSI) for this purpose [10]. The 30-second Chair Stand Test (CST), 40-meter Fast-Paced Walk Test, and Stair Climb Test (SCT) were listed as the three minimal core set of performance-based tests; the 6-Minute Walk Test (6MWT) and Timed Up and Go were listed as noncore tests. Outcome variables from the CST have since been found to be significant predictors of pain, self-reported disability, fall risk, and decreased lower-extremity strength in osteoarthritis patients [11,12]. Similarly, strong associations have been found between the SCT, self-reported walking limitation, and quadriceps and hamstring strength [13]. The simplicity of both the CST and SCT makes possible testing in both clinical and residential settings.

There is precedent in the use of iPhones to quantify outcome variables during the 6MWT in congenital heart failure patients [14]. The ubiquity of iPhones and their built-in sensors may allow for the widespread extrapolation of this utility to the CST and SCT. The primary outcome variables of (1) CST: total number of sit-to-stand cycles and (2) SCT: total ascent and descent duration must be captured by iPhones for this extrapolation to be successful [11,12,15].

Our long-term proposal is to develop an iOS app accessible to the world population, with the ability to instruct osteoarthritis patients in any setting through the CST and SCT, capture clinical outcome data, and export results to clinicians and researchers. As a precursor to this, we developed a prototype app to demonstrate proof of concept and evaluated two iOS sensors of interest: the linear accelerometer and the gravity sensor. We discuss in this paper our experience working with these sensors and report on the criterion validity and test-retest reliability of CST and SCT outcome variable data captured by the app as compared to gold-standard measurements during each test.

## Methods

### iPhone Sensor Triaxial Alignment and Phone Placement

**Figure 1:**
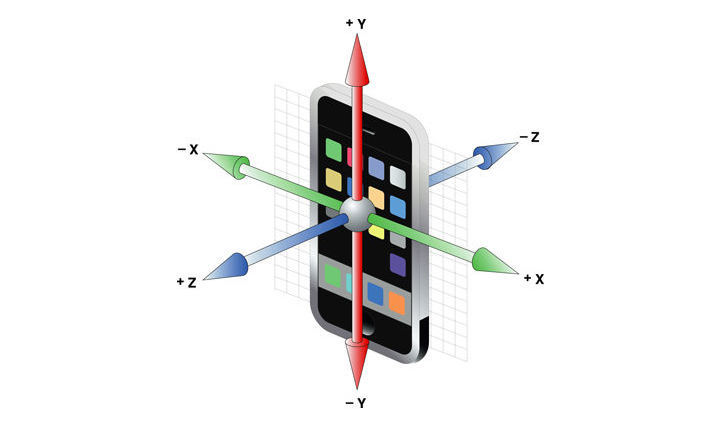
A 3D depiction of an iPhone’s triaxial coordinate system. Notice that both axis and direction are contingent on the phone’s orientation during movement.

The iPhone accelerometer has a preprogrammed triaxial alignment, as depicted in Figure 1. Standing up out of a chair during CST is considered Z acceleration in the 3D-world coordinate system, but the phone may experience X, Y, Z, or a combination of accelerations depending on its orientation.

**Figure 2:**
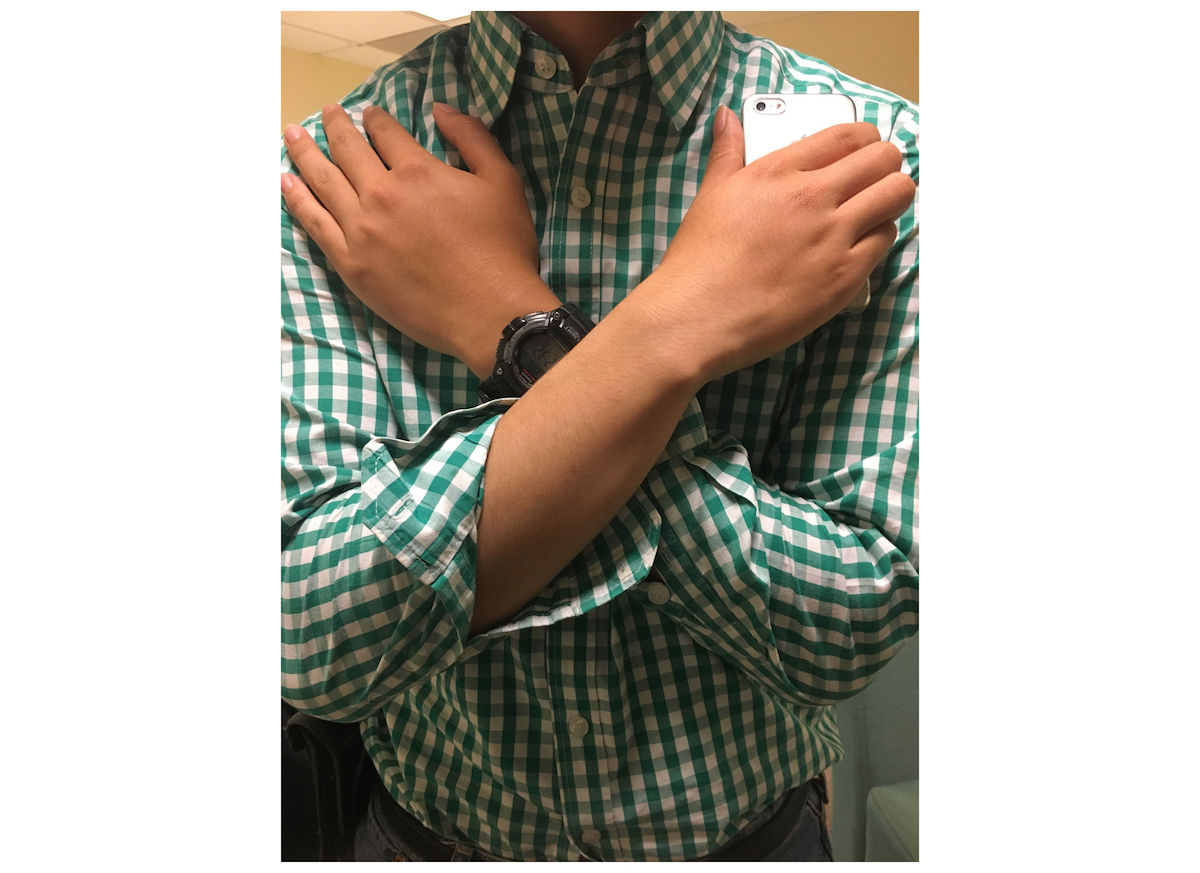
A representation of the phone’s orientation and location during Chair Stand Test testing. An iPhone with its screen facing inward and home button downward would experience +Y acceleration to represent upward vertical movement and –Z acceleration to represent the subject moving forward during the postural transition of standing.

The CST protocol requires subjects to cross their arms across their chest as they stand up out of a chair, so we opted to conduct testing at an analogous phone location and orientation: holding the phone vertically in hand against the chest with arms crossed (see Figure 2).This placement gives Y acceleration peak output during each stand and would make widespread CST testing a practicality, without a requirement for extra equipment.

The gravity sensor was used for the SCT because of its ability to sense changes in gravitational moments along the three axes. We decided to place the phone in the subject’s pants pocket during the SCT. When the subject is standing upright, the phone is vertical in the pocket and feels gravity on its Y-axis; but as the leg is lifted to take the first step, the phone becomes horizontal and the phone feels gravity on its Z-axis, with a simultaneous decrease to zero on the Y-axis. We sought to isolate these features during the SCT to determine the primary outcome variable, total duration of the test. Validation of data obtained at this pocket location could make widespread testing of the SCT a practicality as well.

### Participant Recruitment

Patients were recruited from the University of California San Francisco (UCSF) Center of Research Translation for the Study of Osteoarthritis. Healthy controls were recruited internally from the Musculoskeletal Quantitative Imaging Research Group at UCSF. Sample size was targeted to be a number between 24 and 50 based on recommendations for pilot studies in clinical research [16,17]. Exclusion criteria for controls were confounders that, in the opinion of the tester, could affect the postural transition of standing up (eg, lower extremity orthopedic conditions, morbid obesity, and pregnancy). Patients and controls consented after the study was ethically approved by the UCSF Human Research Protection Office and Institutional Review Board.

### Prototype App Development and Functionality

The prototype app was designed with Swift 3.0 on the XCode 8.3.3 platform (Apple Inc). After using an existing app on the iOS app store that graphically displayed accelerometer and gravity sensor data to determine the optimal phone placement and sensor data collection, we integrated relevant sensor data collection into a user-friendly app with account capabilities and testing prompts to allow for longitudinal participation.

We used the Core Motion framework (Apple Inc) to give the app the ability to collect raw accelerometer data reflecting phone motion. We used FirebaseDatabase and FirebaseAuth frameworks (Google Inc) to set up data storage in Google Firebase, a cloud-hosted real-time database that can facilitate user authentication for iOS apps.

Subject identifier fields requested in the app included the following: *Name*, *Date of Birth*, *Email*, *Password*, and *Subject Classification* (Osteoarthritis, Femoroacetabular Impingement, and Healthy Control). In addition, subjects were prompted prior to the Stair Climb Test to choose the number of steps they will ascend and descend from a range of 8-15 steps.

The 30-second data recording period of the CST was started using a toggle button, with a 3-second latency to allow the subject to cross their arms and orient the phone. This latency is coupled with a countdown on the screen and beeps of various tones to signal the inception of the 30-second countdown.

The recording period of the SCT was also initiated with a toggle button and a 3-second latency to allow the subject to put the phone in their pants pocket, coupled with the same visual and audial cues. The recording period was set to be 45 seconds, with an option to take the phone out of the pocket and end data collection if the test was completed early. Sampling rate was set constant at 100 Hz for both tests, but small fluctuations were observed in the sampling frequency between 100 Hz and 101 Hz as the app collected data.

### Performance Testing Protocol

Testing was conducted in various sites at the UCSF Departments of Orthopaedic Surgery and Radiology.

#### Chair Stand Test

Prior to the CST, a chair of 18 inches in height was placed against a wall to ensure stability. The tester provided an iPhone 6 with the prototype app and requested subjects to hold the phone vertically in their right hand during testing, with the home button facing down and screen facing inward. Subjects were asked to repeat cycles of standing up and sitting down completely at a self-selected speed for 30 seconds during the test.

Each subject pressed the start button and positioned the phone quickly across the chest during the 3-second latency period. At the sound of the start buzzer, subjects began standing and sitting cycles, with the tester manually counting the total number of sit-to-stand cycles. An end buzzer from the app signaled the end of data collection. The entire protocol was repeated for two trials in all subjects. An optional resting period was given between trials as required to reduce fatigue as a confounder. Upon completion of both trials, data were automatically pushed to Google Firebase in real time as JSON files.

#### Stair Climb Test

A stairway of 12 steps was located at each of the UCSF testing locations. The tester provided an iPhone with the prototype app and instructed each subject to choose the leg they planned to take their first step with. Subjects were requested to climb the steps and descend at a self-selected speed.

Each subject pressed the start button and placed the phone in the pocket of the leg they chose to take their first step with. At the sound of the start buzzer, the tester started a stopwatch and the subject proceeded to ascend the stairs. At the end of the descent, after both feet had touched the base of the steps, the stopwatch was stopped. The subject was instructed to take the phone out of their pocket and manually end data collection if the test took less than 45 seconds. The entire protocol was repeated for two trials in all subjects, with a 120-second resting period preset in the app between trials as required. Data was automatically pushed to Google Firebase after completion of the test.

### Data Processing

#### Overview

After exporting the raw data from each test out of Google Firebase in a JSON file format, we used an online conversion tool to change the file format from JSON to CSV. The data from each test were then imported into MATLAB (The MathWorks, Inc) for data processing.

#### Chair Stand Test

In MATLAB, graphs of acceleration in the Y-axis were phase-shifted by subtracting the mean to compensate for the effects of gravity and time-normalized to exactly 3000 frames (30 seconds at 100 Hz) to remediate variation in data collection frequency. A 3 Hz low-pass, fourth-order Butterworth filter and magnitude thresholds were applied to eliminate extraneous peaks due to recoil noise.

After completion of graph processing, local extrema were automatically identified, using a threshold of 0.4 m/s^2^, and labeled in MATLAB as sit-to-stand postural transitions of the individual. The total number of repetitions identified for each trial was juxtaposed with manual counts from testing.

#### Stair Climb Test

The data were phase-shifted in MATLAB by subtracting the mean and a 3 Hz low-pass, fourth-order Butterworth filter was applied to eliminate extraneous data points due to recoil noise. The derivative of each graph was taken to determine change in acceleration. Test graphical features were determined using a threshold of 0.03 m/s^3^ for local extrema. Since a peak in the graph occurred for alternating steps due to the placement of the phone adjacent to one limb, we observed six peaks during the ascent and six during the descent of a 12-step test. The twelfth peak was set as the end marker for the test and the corresponding frame number for this peak was outputted as the outcome variable, test duration, by dividing the frame number by the data collection frequency of 100 Hz.

### Statistical Analysis

#### Validation of Chair Stand Test Outcome Variable: Total Number of Sit-to-Stand Cycles

Observed sit-to-stand count was compared to +Y-acceleration peak count outputted by MATLAB to validate the iPhone’s ability to capture the sit-to-stand feature of each cycle. The average of trials was taken for each subject to obtain average MATLAB count and average manual count for that subject. These values were compared across all subjects to obtain average absolute difference between MATLAB and human counts. Pearson’s correlation coefficient (PCC) was determined in Microsoft Excel between the variables of average MATLAB count and average manual count.

To determine the intraclass correlation coefficient (ICC), the difference between MATLAB and human count for each individual trial was firstly computed using Microsoft Excel. The average of differences across trials was taken for each individual subject to obtain test-retest means. The average and standard deviation of these test-retest means (SDtest-retest) was computed and SPSS Statistics version 23 (IBM Corp) was used to calculate the ICC.

The standard error of measurement (SEM) was calculated as follows:

SEM = SDtest-retest × √(1-ICC) (1)

SEM was then converted into percentage by dividing by the average value of the manual count. SEM less than 10% was set as the threshold for the acceptability of MATLAB calculations [18].

Repeatability coefficients (CRs) were calculated as follows:

CR = 1.96 × SEM × √2 (2)

A Bland-Altman plot with 95% limits of agreement was then created using SPSS to observe the agreement and distribution of differences between app-derived and manual sit-to-stand count.

#### Validation of Stair Climb Test Outcome Variable: Total Ascent + Descent Duration

Stopwatch time of the SCT test was compared to app-derived duration from MATLAB to validate the iPhone’s ability to detect features of the test that enable isolation of the testing interval within the data. Average absolute difference, PCC, SDtest-retest, ICC, and SEM were determined as they were for the CST analysis.

A Bland-Altman plot with 95% limits of agreement was created to evaluate the distribution of differences between app-derived test duration and stopwatch time.

## Results

### Chair Stand Test and Stair Climb Test Graphical Waveforms

**Figure 3:**
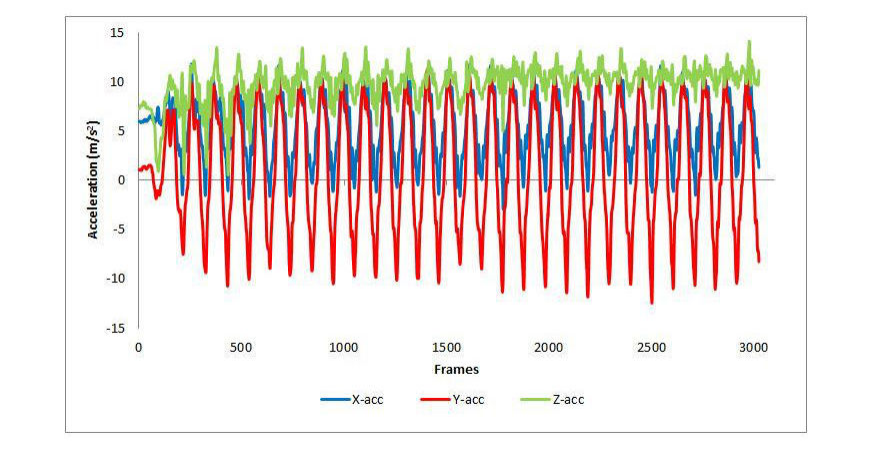
A graphical representation of the 30-second Chair Stand Test as captured by an iOS-based linear accelerometer. Y-axis features represent the postural transitions of sitting and standing and Z-axis features represent anterior and posterior movement during the postural transition. acc: acceleration.

A total of 3 patients (age >50 years; 1 male, 2 female) and 21 healthy controls (age 17-60 years; 12 male, 9 female) participated in testing. Data collection was completely objective and internal controls had a great range of physical capabilities. CST graphical waveforms were characterized by Y peaks and troughs for each sit-to-stand and stand-to-sit repetition, respectively (see Figure 3). Z troughs were observed for the anterior movement during a sit-to-stand repetition and Z peaks for the posterior movement during stand-to-sit repetition. X-axis graphical features were observed if the phone was not held exactly vertical during a trial.

**Figure 4:**
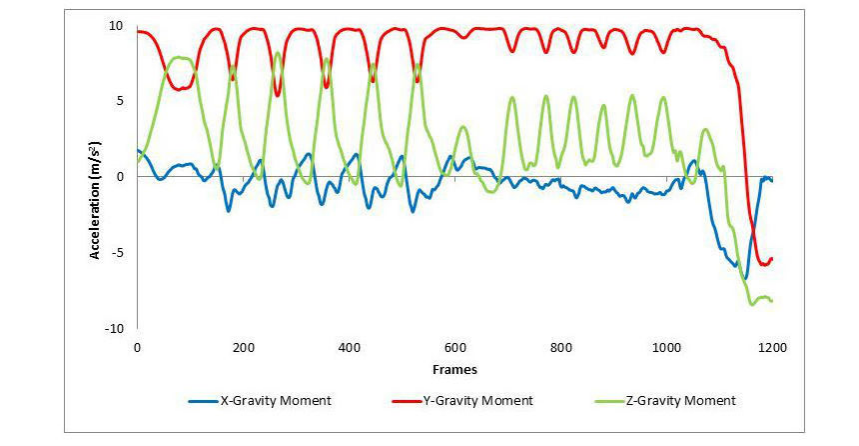
A graphical representation of the Stair Climb Test as captured by an iOS-based gravity sensor. Y-axis features represent changes in gravitational moments during stair ascent and descent.

SCT waveforms contained features exclusive to the stair ascent and descent components of the test (see Figure 4). Each step climbed or descended by the limb adjacent to the phone is seen as a decrease in Y acceleration and an increase in Z acceleration to reflect the change of gravitational moments on the phone. The magnitude of these changes was observed to be greater during the stair ascent than the stair descent.

### App-Derived Chair Stand Test Versus Human-Observed Chair Stand Test

**Figure 5:**
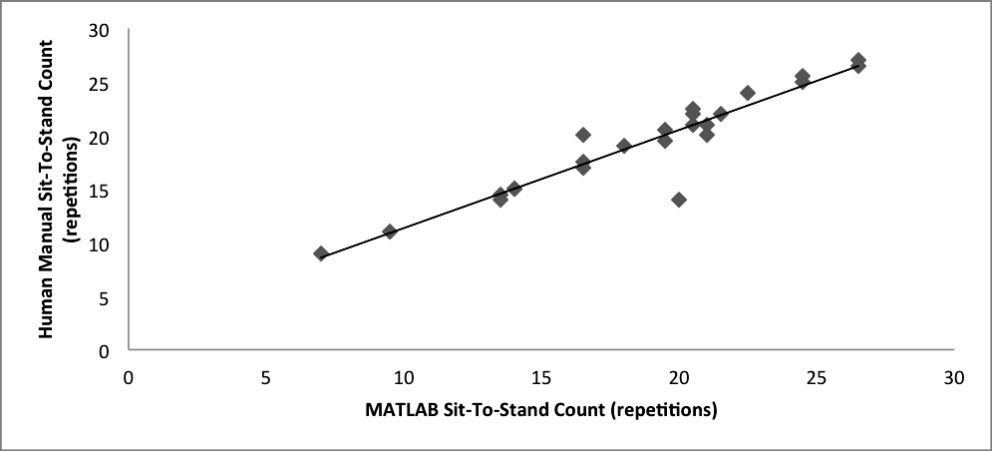
Regression between MATLAB computation and manual count in calculation of 30-second Chair Stand Test repetitions (n=24; Pearson’s correlation coefficient=.890).

A strong Pearson’s correlation (see Figure 5) and intraclass correlation was found between the MATLAB count of Y peaks in the CST graphical data and the human count of stands during testing (see Table 1). The SEM was found to be well within the threshold set for acceptability of MATLAB measurements.

**Figure 6:**
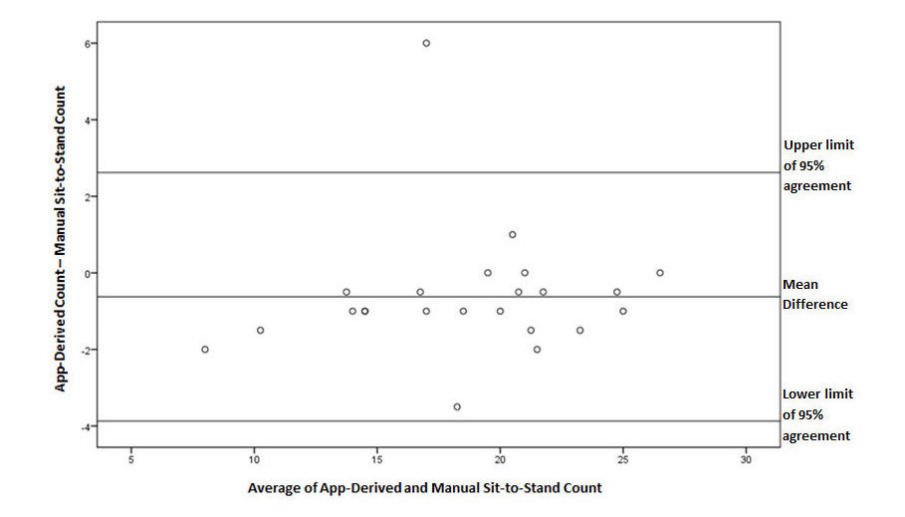
A Bland-Altman plot of differences between app-derived and manual sit-to-stand count during the 30-second Chair Stand Test. Line of mean difference is at -0.63 and upper and lower limits of 95% agreement are at 2.62 and -3.87, respectively.

This concordance and low variance was observed for all subjects regardless of sit-to-stand count. In the Bland-Altman plot, 96% of points fell within the lines of agreement with an even distribution around the line of mean difference at -0.63 (see Figure 6).

**Table 1:** Descriptive statistics of Chair Stand Test and Stair Climb Test with comparisons between app-derived data and human-observed data.

Statistic	Chair Stand Test, stands	Stair Climb Test, seconds
Average human count	19.3	10.9
Average MATLAB count	18.6	9.2
Average absolute difference	0.7	1.7
Pearson’s correlation coefficient	.890	.865
Average test-retest	1.38	1.77
SDtest-retest^a^	1.27	1.32
Intraclass correlation coefficient	.968	.902
Standard error of measurement	0.227	0.433
Standard error of measurement, %	1.12	3.94
Repeatability coefficient	0.629	1.20

^a^SDtest-retest: standard deviation of test-retest means.

### App-Derived Stair Climb Test Versus Stopwatch-Derived Stair Climb Test

**Figure 7:**
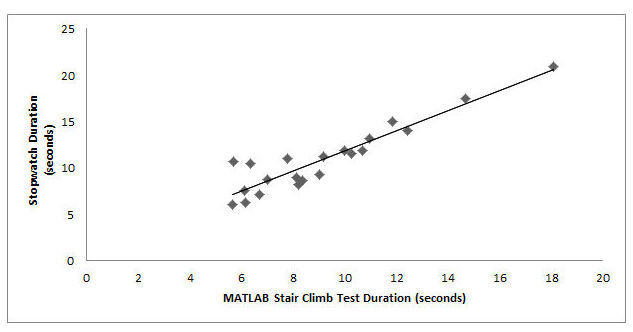
Regression between MATLAB-generated test duration and stopwatch-measured time during the 12-step Stair Climb Test (n=21; Pearson's correlation coefficient=.865).

**Figure 8:**
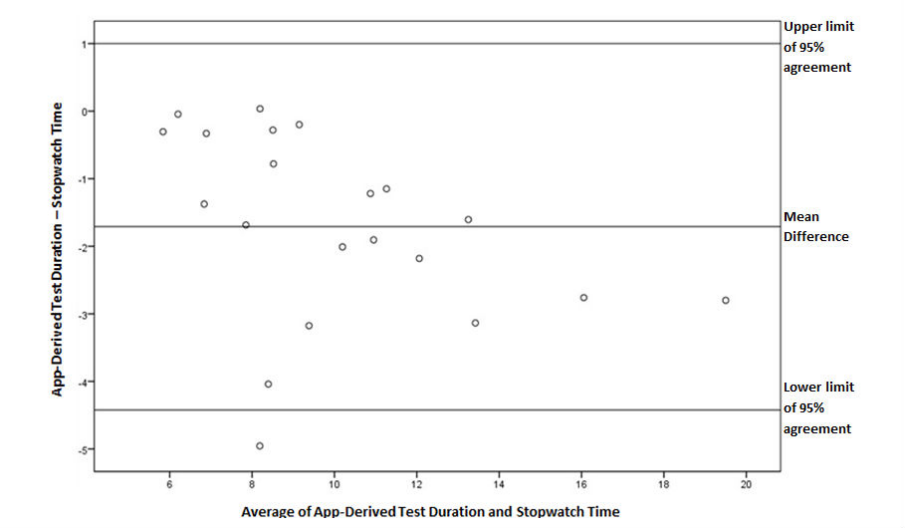
A Bland-Altman plot of differences between app-derived test duration and stopwatch time during the Stair Climb Test. Line of mean difference is at -1.71 and upper and lower limits of 95% agreement are at 1.00 and -4.42, respectively.

A strong Pearson’s correlation and intraclass correlation were observed between MATLAB output test duration and stopwatch-derived duration (see Figure 7 and Table 1). The SEM was within the acceptability threshold and the variance of data appeared to be low between lower and higher test durations (see Figure 7 and Table 1). A total of 95% of data points in the Bland-Altman plot fell within the lines of agreement with a relatively even distribution around the line of mean difference at -1.71 (see Figure 8).

## Discussion

### Principal Findings

Our findings demonstrate that CST and SCT data obtained from iPhone sensors can accurately capture the primary outcome variable from each test. The distinct and consistent data features from each test made it possible to automate data processing using MATLAB. This resulted in very strong values of PCC and ICC and conclusive Bland-Altman plots between manually obtained data and app-derived data, thus establishing criterion validity, test-retest reliability, and agreement. The SEMs for both tests were well below the threshold set in literature, further suggesting the accuracy of our MATLAB algorithm at automating computation of the outcome variables. The CRs further complement the findings of agreement between the app data and the gold-standard manual measurements.

These results have great clinical implication, especially as performance testing using current gold-standard measurements has become a topic of increased interest since OARSI’s recommendations were made public in 2013 [10]. The inefficiency of current testing is often complemented by the fact that the data collected in closed clinical settings may not be representative of a patient’s disability during ADL in their community. There have been previous efforts at the UCSF Department of Cardiology to make remote testing possible utilizing the ubiquity of iPhones [14]. However, the results are confined to one performance test, the 6MWT, and one iPhone tool, the motion sensor. Though the 6MWT is also a part of the battery of five tests suggested by OARSI, it was not listed as a core test and thus was not chosen as a focus of our study.

Our findings on the two core tests of CST and SCT have greater significance in the osteoarthritis community. Recent papers have reported on their relevancy and importance toward tracking clinical markers of lower extremity strength and fall risk, and patient-reported outcomes such as walking limitation, pain, and disability [11-13]. Furthermore, both tests require rapid hip and knee flexion and extension and a larger range of joint motion than walking tests such as the 6MWT. Because these factors tend to be impaired early in osteoarthritis disease progression, longitudinal examination of these tests may help clinicians predict deteriorations and ultimately develop a better care plan [19,20].

Most subjects in our study reported that they would use the app to test longitudinally at home. While the 6MWT-app study included the requirement of a hip holster for one phone placement location, neither of our phone locations require equipment [14]. This adds to the simplicity of our testing protocol and increases the likelihood of acquiring a large sample of subjects from remote testing locations. On the clinical side, automated data processing with our MATLAB codes could make it practical to longitudinally evaluate and track such a large subject sample. Collectively, this could meet the goals of increasing the frequency of assessments in community settings, decreasing the requirement for clinical visits, and providing a streamlined database of longitudinal physical function data from osteoarthritis patients across the world.

### Limitations and Future Directions

Limitations of our study include a low sample size and an overrepresentation of subjects within a younger age group, not well represented in osteoarthritis. Nonetheless, physical abilities varied sufficiently enough to determine iPhone sensor functionality across a large range of outcome variable magnitudes. The SCT in general appeared to be more difficult to accurately capture than the CST, but given that stopwatch times were consistently overestimated compared to MATLAB-derived times, this may be largely due to human reaction time as a confounder in our study.

The absence of at-home testing within our study can also be considered a limitation. Future studies may be required to definitively establish the validity and reliability of at-home app testing in the presence of external raters and the compliance rate of osteoarthritis patients who receive the app to test longitudinally at home without external raters. Should both studies prove to be successful, a large-scale study focused on establishing minimally clinical importance difference for MATLAB-computed counts of CST and SCT in osteoarthritis patients may be warranted.

### Conclusions

An app utilizing the iPhone’s accelerometer and gravity sensor might be a good alternate to accurately and consistently obtain outcome variables during the CST and SCT, respectively. The ease of protocol, lack of adverse events during testing, and ability to automate data extraction collectively suggest the preliminary applicability of iPhones as a safe and reliable tool for widespread and longitudinal performance testing in the future.

## Abbreviations

6MWT: 6-Minute Walk Test
ADL: activities of daily living
CR: repeatability coefficient
CST: Chair Stand Test
ICC: intraclass correlation coefficient
OARSI: Osteoarthritis Research Society International
PCC: Pearson’s correlation coefficient
SCT: Stair Climb Test
SDtest-retest: standard deviation of test-retest means
SEM: standard error of measurement
UCSF: University of California San Francisco
